# Enhanced oxygen reduction reaction on caffeine-modified platinum single-crystal electrodes

**DOI:** 10.1038/s42004-024-01113-6

**Published:** 2024-02-03

**Authors:** Nagahiro Hoshi, Masashi Nakamura, Ryuta Kubo, Rui Suzuki

**Affiliations:** Department of Applied Chemistry and Biotechnology, Faculty of Engineering, Chiba University, 1-33 Yayoi-cho Inage-ku, Chiba, 263-8522 Japan

**Keywords:** Electrocatalysis, Fuel cells

## Abstract

Enhancing the activity of the oxygen reduction reaction (ORR) is crucial for fuel cell development, and hydrophobic species are known to increase the ORR activity. This paper reports that caffeine enhanced the specific ORR activity of Pt(111) 11-fold compared to that without caffeine in a 0.1 M HClO_4_ aqueous solution. Moreover, caffeine increased the ORR activity of Pt(110) 2.5-fold; however, the activity of Pt(100) was unaffected. The infrared (IR) band of PtOH (blocking species of the ORR) decreased for all the surfaces. Caffeine was adsorbed with its molecular plane perpendicular to the Pt(111) and Pt(110) surfaces and tilted relative to the Pt(100) surface. Thus, the effects of caffeine on the ORR activity can be rationalized by a decrease in PtOH coverage and the difference in adsorption geometry of caffeine.

## Abstract (web summary)

Enhancing the activity of the oxygen reduction reaction (ORR) is crucial for fuel cell development, but hydrophobic species used to boost ORR activity are often toxic. Here, the authors report that caffeine enhances the specific ORR activity of Pt(111) eleven-fold compared to that without caffeine in a 0.1 M HClO_4_ aqueous solution, and find that the enhancement mechanism depends strongly on the surface structures of platinum.

## Introduction

A fuel cell is a power generation system with a higher energy conversion efficiency than that of thermal power generation. A major drawback of fuel cells is the high overpotential of the oxygen reduction reaction (ORR) at the air electrode, which causes energy loss in the system. Based on the high ORR overpotential, electrocatalysts in polymer electrolyte fuel cells require high Pt loading. Therefore, an increase in ORR activity decreases the Pt loading required in electrocatalysts.

The combination of well-defined single-crystal electrodes modified with hydrophobic species is an effective method for enhancing the ORR^1^. Markovic et al. reported the structural effects on the ORR on the low-index planes of Pt in 0.1 M HClO_4_: Pt(100) < Pt(111) < Pt(110)^2^. Feliu et al. demonstrated that the ORR activity increases with increasing step atom density using high-index planes of Pt^3,4^. A systematic study of the ORR using the high-index planes of Pt revealed that the (111) terrace edge enhances the ORR activity of Pt electrodes^5^. Markovic et al. reported that Pt oxides are blocking species of the ORR^6^. Infrared reflection absorption spectroscopy (IRAS) and surface-enhanced Raman spectroscopy showed that the ORR activity decreases with the increase of the band intensity of PtOH, verifying that PtOH is the main blocking species of the ORR over Pt single-crystal electrodes^7–9^. Density functional theory (DFT) calculations predicted that the (111) terrace edge degrades the structure of water, decreasing the coverage of blocking species of the ORR, such as PtOH and PtO^10^. The formation of PtOH requires water molecules around the Pt surface, as follows: Pt + H_2_O → PtOH + H^+^ + e^−^. Hydrophobic species expel water around the electrode surface, which may enhance the ORR activity.

Modification with alkyl amines^11,12^, melamine^13,14^, and protonic ionic liquids^15–17^ increases the ORR activity of Pt nanoparticles, as well as those of polycrystalline and single-crystal Pt electrodes. The effects of the hydrophobic species on the ORR activity significantly depend on the surface structure of the Pt electrode. The degree of activity enhancement is the highest on the Pt(111) surface of single-crystal Pt^12,14,16,18^, whereas the Pt(100) surface remains unaffected^14,18^, although it is sometimes deactivated by hydrophobic species^12,16^. Among the hydrophobic species examined, Tetra‒*n*‒hexylammonium cation (THA^+^) has the strongest influence on Pt(111), resulting in an 8-fold enhancement in the ORR activity of Pt(111) compared with that of unmodified Pt(111)^18^.

Caffeine is less toxic than other hydrophobic species, and it activates the hydrogen evolution and oxidation reactions of Pt nanoparticles and caffeine doped carbons^19^. Caffeine is used as a capping agent and a structure directing agent for the preparation of well dispersed Pd–Au nanochain networks with unique structures, and enhances the ORR activity^20^. The ORR activity of caffeine derived graphene-wrapped Fe_3_C nanoparticles is higher than that of Pt/C in alkaline solution^21^. However, the effects of caffeine itself on the ORR have not been examined on single crystal electrodes thus far. In this study, the structural effects of single-crystal Pt electrodes modified with caffeine on the ORR are investigated. In addition, the enhancement mechanism is investigated using IRAS.

## Results and discussion

### Structural effects on the ORR

**Fig. 1:**
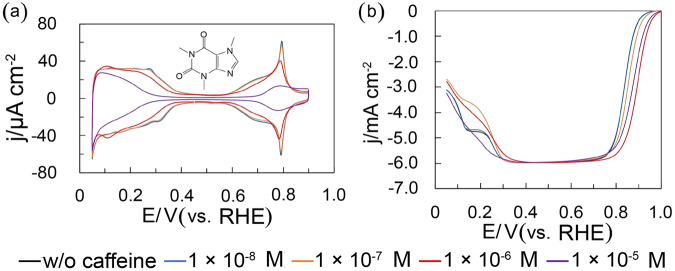
Cyclic voltammograms and linear sweep voltammograms of Pt(111) with and without caffeine in 0.1 M HClO_4_. **a** Voltammograms of the Pt(111) electrode in 0.1 M HClO_4_ saturated with Ar at various caffeine concentrations. Scanning rate: 0.050 V s^−1^. The inset image shows the structure of caffeine. **b** Linear sweep voltammograms of Pt(111) at various caffeine concentrations. Scanning rate: 0.010 V s^−1^. Rotation speed: 1600 rpm. The voltammograms of w/o caffeine overlap those of 1 × 10^−8^ M.

Figure 1a shows the voltammograms of Pt(111) before and after caffeine modification in 0.1 M HClO_4_ saturated with Ar. The charges of the hydrogen adsorption and Pt oxide formation regions decrease with increasing caffeine concentrations. These results show that adsorbed caffeine molecules block the hydrogen adsorption and prevent the Pt oxide formation. Figure 1b shows the linear sweep voltammograms of Pt(111). The cathodic current of the kinetically controlled region between 0.75 and 1.0 V vs. reversible hydrogen electrode (RHE) varies with the caffeine concentration.

**Fig. 2:**
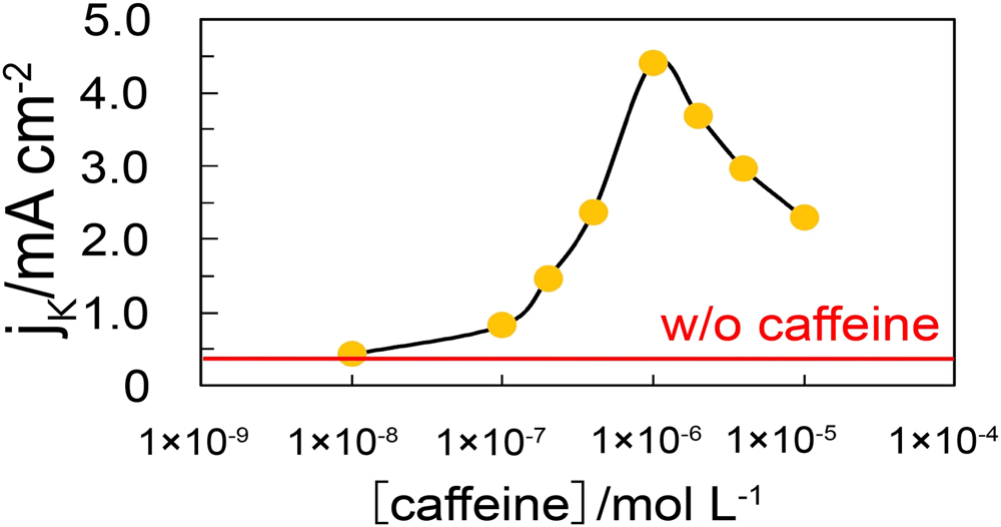
Specific activity of the ORR on Pt(111) plotted against log [*caffeine*]. The red bar represents the activity of Pt(111) without caffeine. Error bars are smaller than marks.

**Fig. 3:**
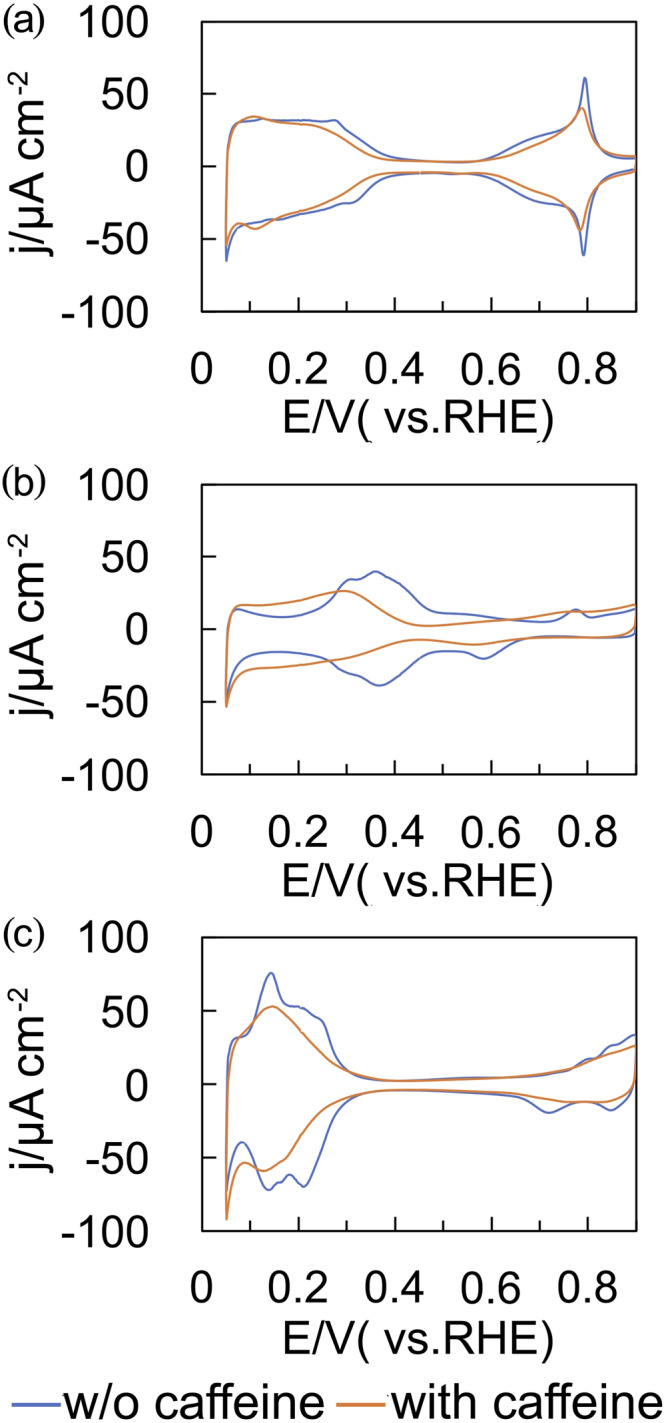
Voltammograms of the low-index planes of Pt in 0.1 M HClO_4_ without caffeine (blue line) and with 1 × 10^−6^ M caffeine (orange line) saturated with Ar. **a** Pt(111), **b** Pt(100), and **c** Pt(110). Scanning rate: 0.050 V s^−1^.

Figure 2 shows the dependence of the specific activity of the ORR (*j*_k_) at 0.90 V(RHE) on caffeine concentration. The *j*_k_ values are significantly dependent on concentration, reaching a maximum at 1 × 10^−6^ M. This *j*_k_ value is 11-fold higher than that obtained in the absence of caffeine, which is the highest among those for the reported hydrophobic species^11–18^. Figure 3 shows voltammograms of the low-index planes of Pt in 0.1 M HClO_4_ containing 1 × 10^−6^ M caffeine, exhibiting the highest *j*_k_ on the Pt(111) surface. The charges of the adsorbed hydrogen and Pt oxide formation regions decrease on all the surfaces after caffeine modification. Decrease of the charge in the adsorbed hydrogen region shows that caffeine molecules block the adsorption sites of hydrogen. However, the increase of *j*_k_ values indicates that the activity for the ORR of unblocked Pt atoms increases by adsorbed caffeine molecules.

Apparent caffeine coverage *θ* was estimated using the charge of “the adsorbed hydrogen region” during the desorption process before (*Q*_H_) and after (*Q*_H_’) caffeine modification using Eq. (1).

1 $$\theta =1 - {{Q}_{{{\rm{H}}}}{\hbox{'}}}/{Q}_{{{{\rm{s}}}}}$$

The *θ* values are 0.18, 0.20, and 0.31 on Pt(111), Pt(100), and Pt(110), respectively, according to the estimation from Fig. 3. Although Pt–OH is present in “the adsorbed hydrogen region” of Pt(100), Pt(110) and stepped surfaces of Pt according to previous reports^8,22–25^, the adsorbed caffeine will block one electron transfer of the Pt–OH formation (Pt + H_2_O → Pt–OH + H^+^ + e^−^) as it prevents the one electron transfer due to the adsorption and desorption of Pt–H. We do not know how many Pt atoms are blocked by one caffeine molecule, but the value of *θ* obtained from Eq. (1) can roughly estimate the ratio of blocked sites by caffeine.

Figure 1S presents linear sweep voltammograms of Pt(111), Pt(110) and Pt(100) modified with caffeine. Limiting current density on Pt(100) decreases markedly below 0.3 V after caffeine modification, showing caffeine promotes H_2_O_2_ formation (two electron transfer). There is a shoulder around 0.8 V in the LSV of Pt(100). The oxide formation around 0.8 V (Fig. 3b) may prevent the ORR on Pt(100).

**Fig. 4:**
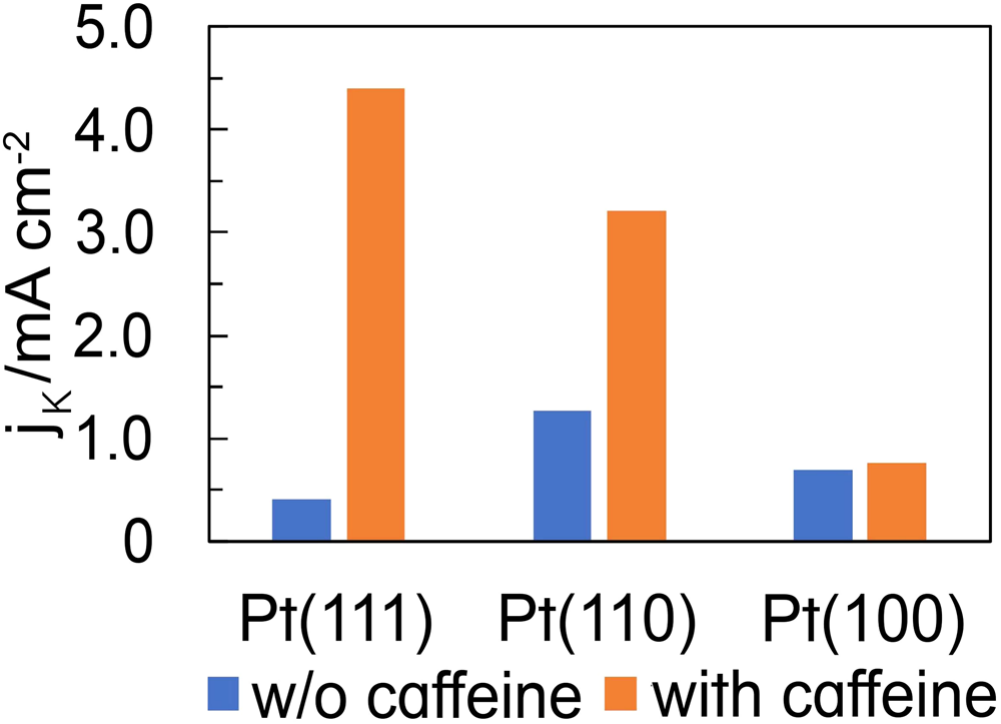
Structural effects on the ORR activity with and without caffeine. The *j*_k_ values at 0.90 V (vs. RHE) on the low-index planes of Pt without caffeine (blue bar) and with 1 × 10^−6^ M caffeine (orange bar) in 0.1 M HClO_4_.

Figure 4 shows the *j*_k_ values before and after the caffeine modification, which increase 11- and 2.5-fold for the Pt(111) and Pt(110) surfaces, respectively. However, the *j*_k_ value for Pt(100) with caffeine is as high as that without caffeine. These results indicate that species other than the Pt oxide affect the ORR activity of single-crystal Pt electrodes modified with caffeine. Therefore, the adsorbed species on the low-index planes of Pt were analyzed using IRAS. Caffeine itself might have the ORR activity. However, no effects of caffeine on the ORR on Pt(100) (Fig. 4) supports that the ORR activity of adsorbed caffeine itself is negligible on Pt electrodes.

### IRAS spectra of adsorbates

When the IRAS spectra were measured, an HF solution was used instead of the HClO_4_ solution because the ClO_4_^−^ band overlaps with the PtOH band. Moreover, F^−^ is IR-inactive, and thus, the PtOH band was detected using IRAS. Fig. S2 shows voltammograms in 0.1 M HF saturated with Ar. Decreases of the charges of the adsorbed hydrogen region in HF after caffeine modification are more significant than those in HClO_4_ (Fig. 3). Caffeine in HF may be more apt to be adsorbed on the low index planes of Pt than that in HClO_4_.

**Fig. 5:**
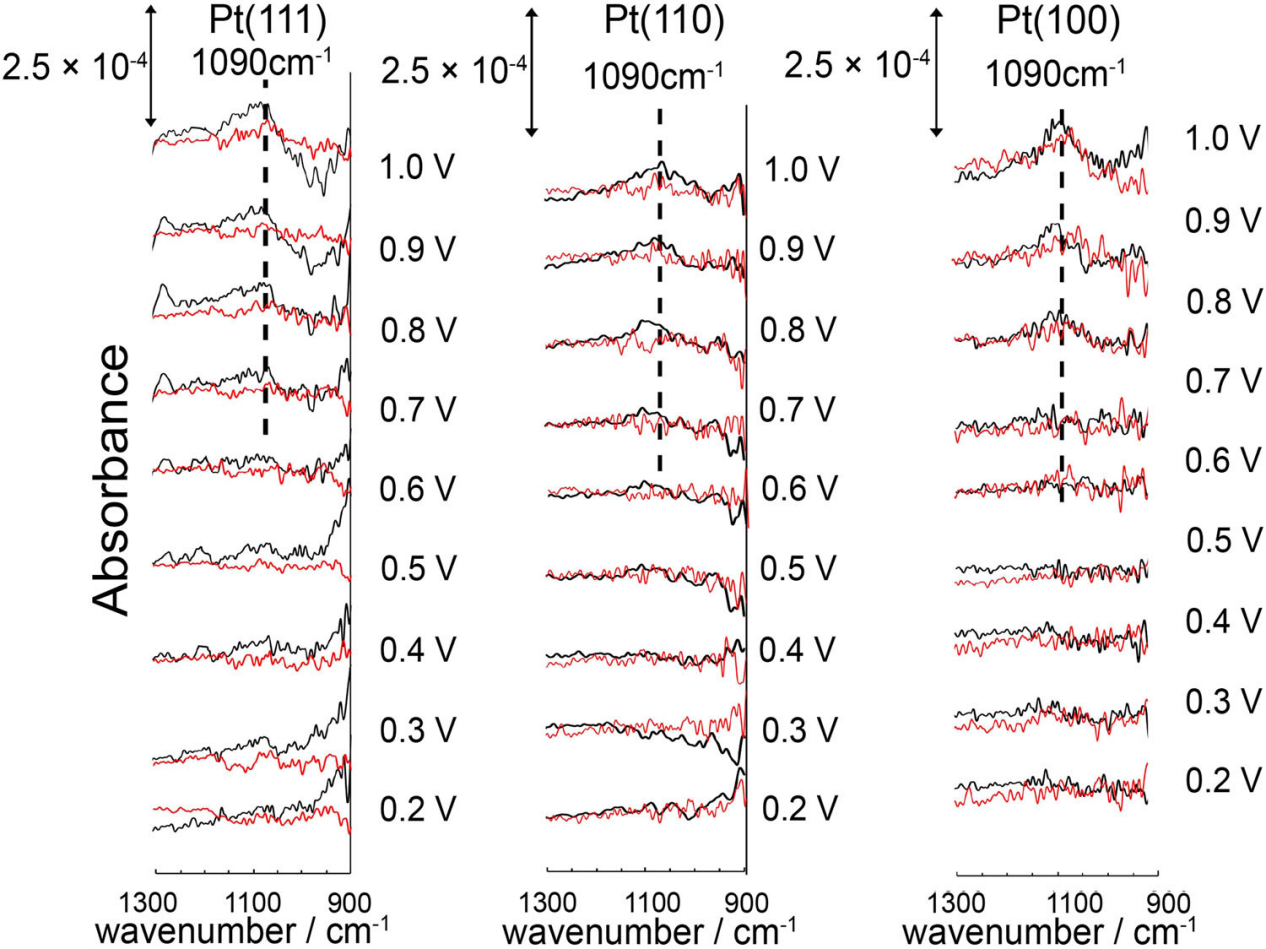
Infrared reflection absorption spectra of the low-index planes of Pt in 0.1 M HF saturated with Ar. Black line: without caffeine. Red line: with 1 × 10^−6^ M caffeine. Reference spectra were collected at 0.1 V (vs. RHE). The spectra were obtained by averaging over 1280 scans with a resolution of 4 cm^−1^.

Figure 5 shows the IRAS spectra between 900 and 1300 cm^−1^ before and after caffeine modification in 0.1 M HF saturated with Ar. A positive band can be observed at ~1090 cm^−1^, which is assigned to the in-plane bending vibration of Pt–O–H *δ*(OH)^8,25^. The band intensity of *δ*(OH) decreases for all the surfaces after caffeine modification. The percentage reduction of the integrated band intensities of *δ*_OH_ for Pt(111) and Pt(110) are 80% and 50%, respectively. Therefore, the increased *j*_k_ values of Pt(111) and Pt(110) after caffeine modification may be attributed to the decrease in PtOH coverage. However, for Pt(100), with an ORR activity unaffected by caffeine, the δ_OH_ band intensity also decreases by 15% after modification. These results demonstrate that PtOH, caffeine, and water affect the ORR of single-crystal Pt electrodes.

The band intensity of IR spectra depends on the direction of the dipole moment as well as the coverage. Adsorbed caffeine may alter the dipole moment direction of δ(OH), causing the decrease of the band intensity of δ(OH) after caffeine modification. However, the charges of the Pt oxides formation decrease on all the surfaces after caffeine modification as shown in Fig. 3, showing that the coverage of PtOH decreases.

**Fig. 6:**
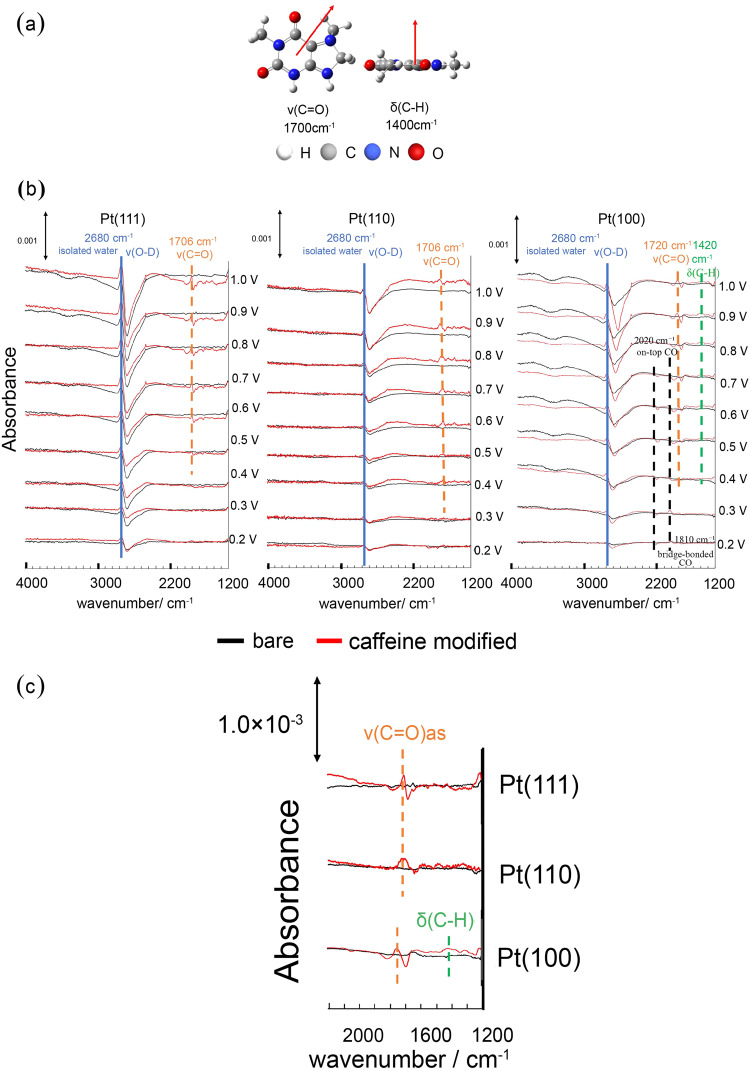
IRAS spectra of the low index planes of Pt with and without caffeine. **a** Dipole moments (red arrow) of the caffeine vibrational modes *ν*(C = O) and *δ*(C–H) calculated using DFT. **b** Infrared reflection absorption spectra on the low-index planes of Pt in 0.1 M HClO_4_/D_2_O saturated with Ar. **c** Magnification of the spectra between 1200 and 2200 cm^−1^. Black line: without caffeine. Red line: with 1 × 10^−6^ M caffeine. Reference spectra were collected at 0.1 V (vs. RHE). The spectra were obtained by averaging over 1280 scans with a resolution of 4 cm^−1^.

According to the surface selection rule of IRAS^26^, a vibrational mode in which the dipole moment is parallel to the electrode surface is IR-inactive, because the dipole moment is negated by the mirror image dipole moment induced in the electrode. In contrast, the vibrational mode in which the dipole moment is vertical or tilted relative to the electrode surface is IR active. According to DFT calculations of caffeine, the dipole moments of the C = O stretching vibration ν(C = O) and C–H bending vibration δ(C–H) are parallel and vertical, respectively, relative to the caffeine molecular plane (Fig. 6a). These bands were used to estimate the adsorption geometry of caffeine on the single-crystal Pt electrodes.

Figure 6b shows the IRAS spectra of the low-index planes of Pt before and after caffeine modification in 0.1 M HF/D_2_O. A D_2_O solution was used instead of H_2_O to prevent the overlap of the vibrational bands of caffeine and H_2_O, and consequently the O–H bending vibrational band of H_2_O (~1600 cm^−1^) is shifted to ~1100 cm^−1^, where no caffeine band occurs.

The positive band at 2680 cm^−1^ may be assigned to the O–D stretching vibration ν(O–D) of isolated water, and the negative band at ~2600 cm^−1^ may be attributed to weakly hydrogen-bonded water ν(O–D)^27–29^. The ν(O–D) band intensity of isolated water increases after caffeine modification, indicating that caffeine degrades the hydrogen-bonding network of D_2_O. The bands associated with isolated water and weakly hydrogen-bonded water form bipolar bands, which complicates the quantitative discussion of their band intensities. However, the increase in the band intensity of ν(O–D) for isolated water is the highest after caffeine modification, although the intensity of the negative band is the largest. This result indicates that the degree of degradation of the hydrogen-bonded network of D_2_O is the highest for Pt(111). The degraded hydrogen-bonding network destabilizes PtOH, as predicted by DFT calculations^10^, resulting in a significant decrease in the PtOH band intensity of Pt(111).

**Fig. 7:**
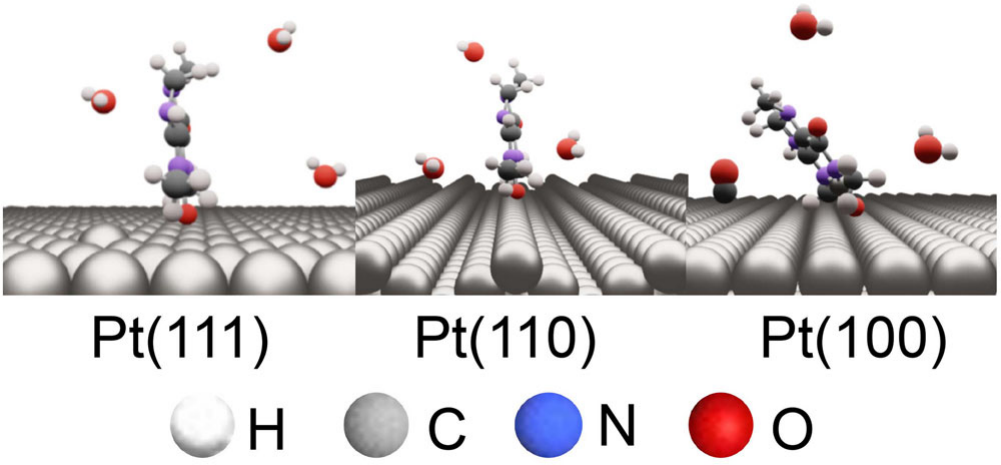
Geometries of adsorbed caffeine on the low-index planes of Pt. Large dark gray, light gray, small dark gray, blue and red spheres show Pt, hydrogen, carbon, nitrogen and oxygen atoms, respectively.

Considering the IRAS bands of caffeine, *ν*(C = O) can be observed at ~1700 cm^−1^ above 0.5 V (vs. RHE), but δ(C–H) is not detected for Pt(111) and Pt(110). This result indicates the adsorption of caffeine onto Pt(111) and Pt(110), with its molecular plane vertical to the electrode surface (Fig. 7). The *ν*(C = O) band shifts to higher wavenumbers with increasing applied potential, indicating that caffeine is adsorbed onto the surface at the carbonyl group. The *ν*(C = O) band is bipolar, and consequently caffeine is also vertically adsorbed onto Pt(111) and Pt(110) at 0.1 V(vs. RHE), where the reference spectra were collected. In contrast, both *ν*(C = O) and *δ*(C–H) can be observed for Pt(100) above 0.5 V (vs. RHE), indicating that caffeine is adsorbed onto Pt(100) with its molecular plane tilted relative to the surface (Fig. 7). As mentioned previously, the bipolar *ν*(C = O) band results in caffeine adsorption on Pt(100) at 0.1 V (vs. RHE). However, *δ*(C–H) is monopolar, and thus *δ*(C–H) is IR-inactive at 0.1 V (vs. RHE). Based on these results, caffeine is adsorbed onto Pt(100) with its molecular plane perpendicular to the surface from 0.1 to 0.4 V (vs. RHE) and tilted relative to the surface above 0.5 V (vs. RHE). Although the band intensity of *δ*(C–H) is small, we obtained reproducible results; the band of *δ*(C–H) is not the noise. Other bands can be observed at ~2020 and ~1810 cm^−1^ on Pt(100) between 0.2 and 0.7 V (vs. RHE). However, DFT calculations exhibited no caffeine bands at these frequencies. Therefore, these bands are assigned to the on-top and bridge-bonded CO produced by partially dissociative caffeine adsorption^30^. Adsorbed CO is a poisoning species of the ORR. However, the adsorbed CO does not affect the ORR at 0.9 V (vs. RHE) because the adsorbed CO is completely oxidized at this potential. Moreover, caffeine does not affect the ORR activity of Pt(100) (Fig. 4). Therefore, the steric hindrance caused by the tilted adsorption geometry of caffeine may counteract the enhancement of the ORR activity achieved by decreasing PtOH.

We do not know why the ORR activity on Pt(111) depends on caffeine concertation markedly as shown in Fig. 2. Nitrogen containing materials such as carbon nitride and N–doped carbon also change the electronic state of platinum, enhancing the catalytic activity^31–33^. The change of the electric state due to the caffeine adsorption may also cause the potential shifts of the peaks of voltammograms in caffeine containing solution (Fig. 3). *In–situ* surface sensitive X-ray absorption spectroscopy (XAS) and X-ray photoelectron spectroscopy (XPS) are necessary for the elucidation of the enhancement mechanism of the ORR by caffeine on single crystal electrodes of Pt.

## Conclusions

The ORR activity on Pt(111) was enhanced 11-fold by modification with caffeine (1.0 × 10^−6^ M). Moreover, caffeine modification of Pt(110) resulted in a 2.5-fold higher ORR activity, but it did not affect the activity of Pt(100). IRAS demonstrated that the band intensity of the PtOH bending vibration *δ*(O–H) decreased by 80%, 50%, and 15% for Pt(111), Pt(110), and Pt(100), respectively, after caffeine modification. The surface selection rule of IRAS indicated that caffeine is adsorbed on Pt(111) and Pt(110) with its molecular plane perpendicular to the surface above 0.1 V (vs. RHE), whereas it is adsorbed on Pt(100) with its molecular plane tilted relative to the surface above 0.5 V (vs. RHE). Therefore, the increased ORR activity of Pt(111) and Pt(110) was attributed to the decreased PtOH coverage and lower steric hindrance of the adsorbed caffeine. Conversely, for Pt(100), the effect of decreasing PtOH was counteracted by the steric hindrance of the adsorbed caffeine, and thus caffeine did not affect the ORR activity.

## Methods

A Pt single-crystal bead was prepared by melting one end of a 1-mmφ Pt wire (99.99% purity) using a H_2_/O_2_ flame, and the melted Pt was solidified slowly^34^. The crystal was oriented to a certain Miller index using the reflection beam of a He–Ne laser from the (111) and (100) facets on the bead^35^, and it was mechanically polished to a mirror finish using a diamond slurry. The polished single crystal was annealed at ~1600 °C in a H_2_/O_2_ flame to remove the distortion caused by the mechanical polishing. Then it was cooled to 25 °C in an Ar/H_2_ (volume ratio = 95/5) atmosphere to obtain an atomically flat surface. The crystal surface was protected using ultrapure water during the transfer to the electrochemical and IRAS cells.

The electrolytic solution was prepared using HClO_4_ (60%, ultrapure, Kanto Chemical), HF (48%, ultrapure, Kanto Chemical), ultrapure water treated with a Milli-Q Advantage A10 instrument (Millipore), and D_2_O (99.8%, Fujifilm Wako Pure Chemical). Caffeine (98.5% purity, special grade) was purchased from Fujifilm Wako Pure Chemicals. We added prescribed amount of 5.0 × 10^−4^ M of caffeine solution to 0.1 M HClO_4_ solution using a micropipette. Caffeine effects on voltammograms, linear sweep voltammograms and IRAS spectra were measured in 0.1 M HClO_4_ containing caffeine. Caffeine is adsorbed on a Pt single crystal electrode as soon as the electrode surface is dipped in 0.1 M HClO_4_ containing caffeine.

Voltammograms and linear sweep voltammograms were measured in a hanging meniscus configuration using an electrochemical analyzer (ALS701CH, BAS) and rotating ring-disk electrode (RRDE)-3 (BAS). The potentials were referenced to an RHE. ORR activity was measured using a hanging meniscus rotating disk electrode at a rotation speed of 1600 rpm. The electrode potential was scanned positively from 0.05 V (vs. RHE) at a scanning rate of 0.010 V s^−1^. The value of *j*_k_ was evaluated using the Koutecky‒Levich equation:

2 $$1/j={1/j}_{{{{\rm{k}}}}}+{1/j}_{{{{\rm{L}}}}}$$

where *j* and *j*_L_ are the total current density and the limiting current density, respectively.

IRAS was conducted using a Fourier-transform infrared (FTIR) spectrometer (FT/IR6600, JASCO, Tokyo, Japan) with p-polarized light at a resolution of 4 cm^−1^. IR light was incident on the electrode through a BaF_2_ or CaF_2_ prism at an incidence angle of 60°. The solution side of BaF_2_ prism was protected with a polypropylene film to prevent the dissolution in the HF solution when the PtOH spectra were measured. A CaF_2_ prism without a film was used for caffeine measurements. IRAS was conducted using subtractively normalized interfacial FTIR spectroscopy, and the reference spectra (*R*_R_) were collected at 0.1 V (vs. RHE). The sample spectra (*R*_S_) were collected from 0.2 to 1.0 V (vs. RHE) at a resolution of 4 cm^−1^, and the spectra were averaged over 1280 scans. *Absorbance* was calculated using Eq. (3).

3 $$A{bsorbance}=-\log ({R}_{{{{\rm{S}}}}}/{R}_{{{{\rm{R}}}}})$$

DFT calculation was done using Gaussian 16 W. Basis set and density function were 6-31 G** and B3LYP, respectively.

### Supplementary information

Peer Review File

Supplimental Material

Description of Additional Supplementary File

Supplimental Data 1

## Data Availability

The data that support the findings of this study are available from the corresponding author upon reasonable request. All the data are provided as Supplementary Data 1.
