# Supplementary material for: Enhanced oxygen reduction reaction on caffeine-modified platinum single-crystal electrodes
Source: Commun Chem. 2024 Feb 3;7:23. doi: 10.1038/s42004-024-01113-6 (PMC10838267; doi:10.1038/s42004-024-01113-6)
Supplement: Supplementary file 2 — Supplimental Material [file 42004_2024_1113_MOESM2_ESM.pdf]

Supplementary Information

Enhanced oxygen reduction reaction on caffeine-modified Pt single-crystal electrodes

Nagahiro Hoshi*, Masashi Nakamura, Ryuta Kubo, Rui Suzuki

Department of Applied Chemistry and Biotechnology, Faculty of Engineering, Chiba University, 1-33 Yayoi-cho Inage-ku Chiba 263-8522 Japan

E-mail address: hoshi@faculty.chiba-u.jp

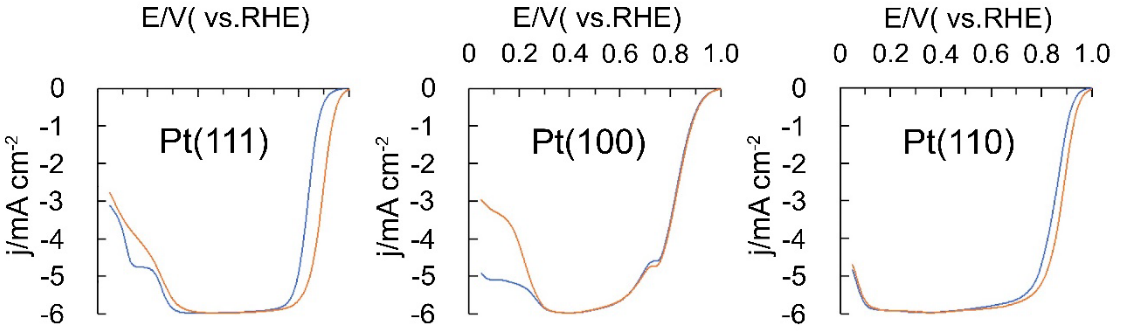

Fig. S1 Linear sweep voltammograms of the low index planes of Pt in 0.1 M $HClO_4$ saturated with O_2. Blue line: without caffeine, orange line: 1×10^{-6} M caffeine containing solution.

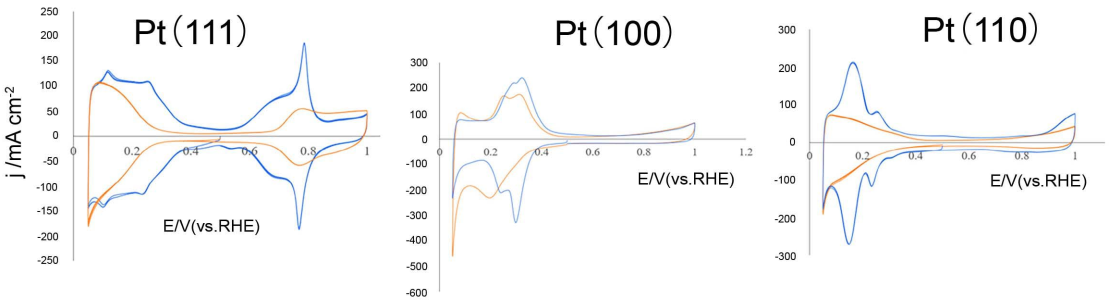

Fig. S2 Voltammograms of the low index planes of Pt in 0.1 M HF saturated with Ar. Blue line: without caffeine, orange line: 1×10^{-6} M caffeine containing solution.
