# Supplementary material for: Enhanced oxygen reduction reaction on caffeine-modified platinum single-crystal electrodes
Source: Commun Chem. 2024 Feb 3;7:23. doi: 10.1038/s42004-024-01113-6 (PMC10838267; doi:10.1038/s42004-024-01113-6)
Supplement: Supplementary file 3 — Description of Additional Supplementary File [file 42004_2024_1113_MOESM3_ESM.pdf]

Description of Additional Supplementary Files

File name: Supplementary Data 1

Description:

Numerical source data of voltammograms (Figs.1, 3), concentration dependence of caffeine on the ORR activity (Fig. 2), structural effects on the ORR activity (Fig. 4), IRAS spectra of PtOH (Fig. 5) and caffeine (Fig. 6).
