# Supplementary material for: Enhanced oxygen reduction reaction on caffeine-modified platinum single-crystal electrodes
Source: Commun Chem. 2024 Feb 3;7:23. doi: 10.1038/s42004-024-01113-6 (PMC10838267; doi:10.1038/s42004-024-01113-6)
Supplement: Supplementary file 1 — Peer Review File [file 42004_2024_1113_MOESM1_ESM.pdf]

nature portfolio

Peer Review File

Enhanced oxygen reduction reaction on caffeine-modified platinum single-crystal electrodes

Open Access This file is licensed under a Creative Commons Attribution 4.0 International License, which permits use, sharing, adaptation, distribution and reproduction in any medium or format, as long as you give appropriate credit to the original author(s) and the source, provide a link to the Creative Commons license, and indicate if changes were made. In the cases where the authors are anonymous, such as is the case for the reports of anonymous peer reviewers, author attribution should be to 'Anonymous Referee' followed by a clear attribution to the source work. The images or other third party material in this file are included in the article's Creative Commons license, unless indicated otherwise in a credit line to the material. If material is not included in the article's Creative Commons license and your intended use is not permitted by statutory regulation or exceeds the permitted use, you will need to obtain permission directly from the copyright holder. To view a copy of this license, visit http://creativecommons.org/licenses/by/4.0/.

Note: Parts of this Peer Review File have been redacted as indicated to maintain the confidentiality of unpublished data.

Reviewers' comments:

Reviewer #1 (Remarks to the Author):

This manuscript reports on the modification of Pt single crystals with caffeine to enhance their oxygen reduction reaction activities. It was found that caffeine enhanced the specific ORR activity of Pt(111) 11-fold compared to that without caffeine. Moreover, caffeine increased the ORR activity of Pt(110) 2.5-fold; however, the activity of Pt(100) was unaffected. The topic and results are interesting. However, some important data are missing. I recommend a major revision is required before the consideration of the manuscript for publication.

1. There are have been some papers talking about caffeine doped carbons, or caffeine treated Pt/Au catalysts for ORR (RSC Adv., 2014,4, 52640-52646), which should be included and discussed in the introduction section.
2. The method of caffeine modification is not introduced and described in the experimental section.
3. Images, such as SEM, showing the morphology of the Pt electrodes before and after the caffeine modification should be provided.
4. Some reports mentioned that caffeine itself also shows some ORR activity. Will the caffeine show some ORR activity in the present work?
5. The loading amount of caffeine on different Pt planes should be provided.
6. Will caffeine modification affect the stability of Pt for ORR? Results should be provided with related comments and discussions.
7. Currently, Pt nanoparticles, rather than Pt plate, are widely used for ORR. The authors should provide the results of caffeine modification of Pt nanoparticles for ORR.

Reviewer #2 (Remarks to the Author):

The authors should discuss the electrochemistry in more detail. The electrochemistry is not discussed enough, differences among surfaces in activity or CVs are barely addressed. It is not clear if what the authors see is simply caffeine blocking Pt active sites that block any adsorption on the surface.
Specific comments:
1. The OHads peak shifts to more negative potentials with 1x10-7-1x10-6 M caffeine. Shifts to more negative potentials have been reported to be due to a stabilisation of the OHads on the surface. This shift can be observed in the voltammogram of Fig 1 and in all CVs in Fig. 3. Can the authors discuss more about this shift?
2. I don’t understand why the study has been performed on the low-index planes of Pt. If the reason is to correlate terrace vs. step phenomena, then surfaces with larger terraces (>3 atoms wide) need to be chosen. Explain why those Pt{hkl} orientations were picked.
3. Specify why the charge decreases after caffeine addition. Is caffeine blocking active sites on the surface?
4. Caffeine coverage estimation through charge before and after modification is not an accurate method, given that the authors later prove that the orientation of caffeine on the surface varies depending on the surface geometry. Also, OH is present on the Pt surface below 0.4 V and is responsible for the sharp

Hupd peak of Pt by exchanging with Had, as proposed by Koper and his colleagues (see also Rizo, Rubén, et al. "Investigating the presence of adsorbed species on Pt steps at low potentials." Nature communications 13.1 (2022): 2550.) ; therefore, it may be imprecise to estimate the caffeine coverage using this method.
5. On page 6, last paragraph, authors talk about "other species other than Pt oxide". However PtOH is not typically considered as a Pt oxide species which appear at potentials above 0.9 V vs RHE for Pt(111).
6. Why does the lowest coverage (for Pt111 vs other surfaces) give the highest activity?
7. Why is the activity highest at 10-6 M and not 10-5 M? What happens at higher concentrations?
8. To further demonstrate that HF could be used instead of HClO4, the authors should provide the CV and ORR test in 0.1M HF before and after caffeine deposition. For the same reason, CV and ORR in D2O should also be given.
9. In IRAS measurements, the band intensity is also dependent on the incident angle of IR light and the orientation of adsorbed surface. Theoretically, OHad adsorbed at the Pt atop site with Pt-O axis perpendicular to surface therefore the band intensity of δPtOH could be proportional to the coverage of OHad at the same cell geometry. Does caffeine deposition alter this and affect the correlation between band intensity and OH coverage?
10. At 0.9VRHE, the relative band intensity seems to be Pt(111) > Pt(100) > Pt(110) , which is opposite against the ORR activity. However, the IRAS spectra after the caffeine deposition displayed in Figure 5 are of poor quality, is this irrelevant to the OH coverage?
11. Please provide the appropriate citations throughout the paper for certain statements. For example, as the article relies on the IRAS to calculate the OH coverage, the reason should be cited or provided so that readers can more broadlyunderstand the concept; The attribution of peak location of ν(C=O) and δ(C-H); Also surface selection rule of IRAS.
12. The unit of the reference absolute intensity in Figure 5 should be 2.5 x 10-4 instead of 2.5 x 104.

Reviewer #3 (Remarks to the Author):

In the manuscript "Enhanced oxygen reduction reaction on caffeine-modified Pt single-crystal electrodes", the authors reported that caffeine increases the specific ORR activity of Pt(111) single-crystal electrodes by 11-fold and that of Pt(110) by 2.5-fold compared to caffeine-free Pt. The increased ORR activity of Pt(111) and Pt(110) can be attributed to the decreased PtOH coverage and lower steric hindrance of the adsorbed caffeine. However, before this manuscript could be considered for publication, some main issues must be well addressed. The specific comments are as follows.
1. There is an obvious error in Figure 1, where no "without caffeine" data comparison line occurs.
2. It is necessary to further supplement the schematic diagram of the synthesis process.
3. The authors described "The charges of the hydrogen adsorption and Pt oxide formation regions decrease with increasing caffeine concentrations." It is necessary to further explain the reason.
4. It is essential to supplement the LSV curves of caffeine-modified Pt(111), Pt(110), and Pt(100) single-crystal electrodes. In addition, Pt/C should be introduced for performance comparison.
5. The authors described " Fig. 4 shows the jk values before and after the caffeine modification, which increase 11- and 2.5-fold for the Pt(111) and Pt(110) surfaces, respectively." Why the Pt(111) offers more significant performance improvements than the Pt(110)?
6. The authors described "The surface selection rule of IRAS indicated that caffeine is adsorbed on

Pt(111) and Pt(110) with its molecular plane perpendicular to the surface above 0.1 V (vs. RHE), whereas it is adsorbed on Pt(100) with its molecular plane tilted relative to the surface above 0.5 V (vs. RHE).” To support this point, it is necessary to provide more visual and obvious evidence.

Reply to Reviewers' Comments

Reviewer #1 (Remarks to the Author):

Thank you for your comments to improve our manuscript. Reply to the comments have been listed below. Revised parts have been highlighted in yellow in the manuscript.

(Comment 1)

There are have been some papers talking about caffeine doped carbons, or caffeine treated Pt/Au catalysts for ORR (RSC Adv., 2014,4, 52640-52646), which should be included and discussed in the introduction section.

(Reply)

The reference "RSC Adv., 2014,4, 52640-52646" reports one-pot synthesis and the ORR of Pd/Au alloy. They do not report caffeine effects on the ORR. We also searched caffeine effects on the ORR of Pt/Au catalysts and caffeine doped carbons using Scifinder n, but we could not find any related papers.

(Comment 2)

The method of caffeine modification is not introduced and described in the experimental section.

(Reply)

Following sentences have been added in Method section.

"We added prescribed amount of 5.0×10^{-4} M of caffeine solution to 0.1 M $HClO_4$ solution using a micropipette. Caffeine effects on voltammograms, linear sweep voltammograms and IRAS spectra were measured in 0.1 M $HClO_4$ containing caffeine."

(Comment 3)

Images, such as SEM, showing the morphology of the Pt electrodes before and after the caffeine modification should be provided.

(Reply)

Atomic and molecular images of Pt electrodes and caffeine cannot be obtained using SEM. In-situ STM or FM-AFM is necessary for the studies. It will take several years to complete these studies. Determination of surface morphology of caffeine modified Pt single crystal electrodes is our next target of our study.

(Comment 4)

Some reports mentioned that caffeine itself also shows some ORR activity. Will the

caffeine show some ORR activity in the present work?

(Reply)

Following sentence is added in the second paragraph of page 7.

"No effects of caffeine on the ORR activity on Pt(100) also supports that the ORR activity of caffeine itself is negligible."

(Comment 5)

The loading amount of caffeine on different Pt planes should be provided.

(Reply)

The ORR activities of different Pt planes were compared in 0.1 M $HClO_4$ containing 1×10^{-6} M caffeine as shown in Fig. 4. Coverages of caffeine are 0.18, 0.20, and 0.31 on Pt(111), Pt(100), and Pt(110), respectively. These values are shown in the second paragraph of page 5.

(Comment 6)

Will caffeine modification affect the stability of Pt for ORR? Results should be provided with related comments and discussions.

(Reply)

We did accelerated durability test by applying square wave between 0.60 and 1.0 V to Pt(111) at room temperature. The ORR activity of caffeine modified Pt(111) was still 6.2–fold as high as that without caffeine after 5000 cycles. These results will be reported in our forthcoming paper.

(Comment 7)

Currently, Pt nanoparticles, rather than Pt plate, are widely used for ORR. The authors should provide the results of caffeine modification of Pt nanoparticles for ORR.

(Reply)

This study is part of a consortium project. The effect of caffein on the ORR of Pt/C was studied by another group in the consortium. Caffeine also improve the ORR activity of Pt/C. The results will be reported by the another group.

Reviewer #2 (Remarks to the Author):

Thank you for your comments to improve our manuscript. Reply to the comments have been listed below. Revised parts have been highlighted in yellow in the manuscript.

(Comment 1)

The OH_{ads} peak shifts to more negative potentials with $1x10^{-7}$-$1x10^{-6}$ M caffeine. Shifts to more negative potentials have been reported to be due to a stabilization of the OH_{ads} on the surface. This shift can be observed in the voltammogram of Fig 1 and in all CVs in Fig. 3. Can the authors discuss more about this shift?

(Reply)

In Fig. 1, the yellow and red lines show voltammograms of 1×10^{-7} and 1×10^{-6} M caffeine. No negative shift of OH_{ads} peak is found. In Fig. 3, negative shift of OH_{ads} peak is not found on Pt(110) either. On Pt(100) in Fig. 3, we cannot estimate the shift of OH_{ads} peak above 0.7 V(RHE) because the current of the double layer region is tilted in caffeine containing solution. However, OH_{ads} charge in “the adsorbed hydrogen region” decreases in n caffeine containing solution. These results support that caffeine prevent the formation of OH_{ads}.

(Comment 2)

I don’t understand why the study has been performed on the low-index planes of Pt. If the reason is to correlate terrace vs. step phenomena, then surfaces with larger terraces (>3 atoms wide) need to be chosen. Explain why those Pt{hkl} orientations were picked.

(Reply)

We would like to summarize the results of the low index planes earlier. Study on the high index planes is now on progress, and the results will be reported later.

(Comment 3)

Specify why the charge decreases after caffeine addition. Is caffeine blocking active sites on the surface?

(Reply)

Following sentences are added in the first paragraph of page 5.

Decrease of the charge in “the adsorbed hydrogen region” shows that caffein molecules block the adsorption sites of hydrogen. However, the increase of j_k values indicates that the activity for the ORR of unblocked Pt atoms increases by adsorbed caffeine molecules.

(Comment 4)

Caffeine coverage estimation through charge before and after modification is not an accurate method, given that the authors later prove that the orientation of caffeine on the surface varies depending on the surface geometry. Also, OH is present on the Pt surface below 0.4 V and is responsible for the sharp H_{upd} peak of Pt by exchanging with H_{ad}, as proposed by Koper and his colleagues (see also Rizo, Rubén, et al. "Investigating the presence of adsorbed species on Pt steps at low potentials." Nature communications 13.1 (2022): 2550.) ; therefore, it may be imprecise to estimate the caffeine coverage using this method.

(Reply)

Following sentences have been revised in the second paragraph of page 5.

"Apparent caffeine coverage θ was estimated using the charge of "the adsorbed hydrogen region" during the desorption process before (Q_H) and after (Q_H') caffeine modification using Equation (1).

$$\theta = 1 - Q_H'/Q_H \qquad (1)$$

The θ values are 0.18, 0.20, and 0.31 on Pt(111), Pt(100), and Pt(110), respectively, according to the estimation from Fig. 3. Although Pt–OH is present in "the adsorbed hydrogen region"[20], the adsorbed caffeine will block one electron transfer of the Pt–OH formation ($Pt + H_2O \rightarrow Pt\text{–}OH + H^+ + e^-$) as it prevents the one electron transfer due to the adsorption and desorption of Pt–H. We do not know how many Pt atoms are blocked by one caffeine molecule, but the value of θ obtained from equation (1) can roughly estimate the ratio of blocked sites by caffeine."

(Comment 5)

On page 6, last paragraph, authors talk about "other species other than Pt oxide". However PtOH is not typically considered as a Pt oxide species which appear at potentials above 0.9 V vs RHE for Pt(111).

(Reply)

We proved that PtOH is a main blocking species of the ORR on single crystal electrodes of Pt.

T. Ueno, H. Tanaka, S. Sugawara, K. Shinohara, A. Ohma, N. Hoshi, M. Nakamura, "Infrared spectroscopy of adsorbed OH on *n*(111)-(100) and *n*(111)-(111) series of Pt electrode ", *J. Electroanal. Chem.*, 800, 162-166 (2017).
doi: 10.1016/j.jelechem.2016.11.028

(Comments 6 and 7)
Why does the lowest coverage (for Pt(111) vs other surfaces) give the highest activity? Why is the activity highest at 10^{-6} M and not 10^{-5} M? What happens at higher concentrations?
(Reply)
Following sentences are inserted in the second paragraph of page 14.
"We do not know why the ORR activity on Pt(111) depends on caffeine concertation markedly as shown in Fig. 2. Nitrogen containing materials such as carbon nitride and N–doped carbon also change the electronic state of platinum, enhancing the catalytic activity[27-29]. *In–situ* surface sensitive X-ray absorption spectroscopy (XAS) and X-ray photoelectron spectroscopy (XPS) are necessary for the elucidation of the enhancement mechanism of the ORR by caffeine on single crystal electrodes of Pt."

(Comment 8)
To further demonstrate that HF could be used instead of $HClO_4$, the authors should provide the CV and ORR test in 0.1M HF before and after caffeine deposition. For the same reason, CV and ORR in D_2O should also be given.
(Reply)
Following sentences have been inserted in the second paragraph of page 8.
"When the IRAS spectra were measured, an HF solution was used instead of the $HClO_4$ solution because the ClO_4^- band overlaps with the PtOH band. Moreover, F^- is IR-inactive, and thus, the PtOH band was detected using IRAS. Fig. S2 shows voltammograms in 0.1 M HF saturated with Ar. Decreases of the charges of "the adsorbed hydrogen region" in HF after caffeine modification are more significant than those in $HClO_4$ (Fig. 3). Caffeine in HF may be more apt to be adsorbed on the low index planes of Pt than that in $HClO_4$."
A measurement using rotating disk electrode (RDE) is done in the open cell because the working electrode must be rotated. Thus a measurement of linear sweep voltammogram (LSV) using RDE in HF solution is a dangerous game; we did not measure LSV in HF solution.
LSVs in D_2O solution will be different from those in H_2O, because the rate determining step of the ORR on a Pt electrode is predicted to be the proton and electron transfer to the adsorbed O_2 according the theoretical calculations. Elucidation of this prediction is out of scope of this study.

(Comment 9)

In IRAS measurements, the band intensity is also dependent on the incident angle of IR light and the orientation of adsorbed surface. Theoretically, OH_{ad} adsorbed at the Pt atop site with Pt-O axis perpendicular to surface therefore the band intensity of δPtOH could be proportional to the coverage of OH_{ad} at the same cell geometry. Does caffeine deposition alter this and affect the correlation between band intensity and OH coverage?

(Reply)

The following sentences have been inserted in the first paragraph of page 9.

"The band intensity of IR spectra depends on the direction of the dipole moment as well as the coverage. Adsorbed caffeine may alter the dipole moment direction of δ(OH), causing the decrease of the band intensity of δ(OH) after caffeine modification. However, the charges of the Pt oxides formation decrease on all the surfaces after caffeine modification as shown in Fig. 3, showing that the coverage of PtOH decreases."

(Comment 10)

At 0.9 V RHE, the relative band intensity seems to be Pt(111) > Pt(100) > Pt(110) , which is opposite against the ORR activity. However, the IRAS spectra after the caffeine deposition displayed in Figure 5 are of poor quality, is this irrelevant to the OH coverage?

(Reply)

Although the quality of the spectra is low, integrated band intensity of δ(OH) increases as Pt(110) < Pt(111) < Pt(100) as reported in our previous report: *Electrocatalysis*, 6, 295-299 (2015).

(Comment 11)

Please provide the appropriate citations throughout the paper for certain statements. For example, as the article relies on the IRAS to calculate the OH coverage, the reason should be cited or provided so that readers can more broadly understand the concept; The attribution of peak location of ν(C=O) and δ(C-H); Also surface selection rule of IRAS.

(Reply)

We have added the related references. Bands of ν(C=O) and δ(C-H) were assigned using DFT calculation because IR data book does not show the direction of the dipole moment.

(Comment 12)

The unit of the reference absolute intensity in Figure 5 should be 2.5×10^{-4} instead of 2.5×10^{4}.

(Reply)

The unit has been revised to 2.5×10^{-4}. Thank you for the suggestion.

Reviewer #3 (Remarks to the Author):

Thank you for your comments to improve our manuscript. Reply to the comments have been listed below. Revised parts have been highlighted in yellow in the manuscript.

(Comment 1)
There is an obvious error in Figure 1, where no "without caffeine" data comparison line occurs.
(Reply)
There is no error in Fig. 1. The voltammograms of w/o caffeine overlap those of 1×10^{-8} M. Following sentence has been inserted in the caption of Fig. 1.
"The voltammograms of w/o caffeine overlap those of 1×10^{-8} M."

(Comment 2)
It is necessary to further supplement the schematic diagram of the synthesis process.
(Reply)
We did not synthesize any chemicals. All chemicals examined were purchased from reagent manufacturers. Preparation method of a Pt single crystal is well–known, and were described in detail in references 31 and 32.

(Comment 3)
The authors described "The charges of the hydrogen adsorption and Pt oxide formation regions decrease with increasing caffeine concentrations." It is necessary to further explain the reason.
(Reply)
Following sentence has been inserted in the first paragraph of page 4.
"These results show that adsorbed caffeine molecules block the hydrogen adsorption and prevent the Pt oxide formation."

(Comment 4)
It is essential to supplement the LSV curves of caffeine-modified Pt(111), Pt(110), and Pt(100) single-crystal electrodes. In addition, Pt/C should be introduced for performance comparison.
(Reply)
LSVs have been shown in Supporting Information as Fig. S1, and following sentences have been inserted in the second paragraph of page 7.

"Fig. 1S presents LSVs of Pt(111), Pt(110) and Pt(100) modified with caffeine. Limiting current density on Pt(100) decreases markedly below 0.3 V after caffeine modification, showing caffeine promotes H_2O_2 formation (two electron transfer). There is a shoulder around 0.8 V in the LSV of Pt(100). The oxide formation around 0.8 V (Fig. 3(b)) may prevent the ORR on Pt(100)."

We have been studying caffeine effects on the ORR in a consortium project. Another group in the consortium found that caffeine also increases the ORR activity of Pt/C. The result will be reported by the group.

(Comment 5)

The authors described "Fig. 4 shows the j_k values before and after the caffeine modification, which increase 11- and 2.5-fold for the Pt(111) and Pt(110) surfaces, respectively." Why the Pt(111) offers more significant performance improvements than the Pt(110)?

(Reply)

Nitrogen containing materials affect the electronic state of platinum. The effects on Pt(111) may differ from that of Pt(110).

Following sentences are inserted in the second paragraph of page 14.

"We do not know why the ORR activity on Pt(111) depends on caffeine concertation markedly as shown in Fig. 2. Nitrogen containing materials such as carbon nitride and N–doped carbon also change the electronic state of platinum, enhancing the catalytic activity[27-29]. *In–situ* surface sensitive X-ray absorption spectroscopy (XAS) and X-ray photoelectron spectroscopy (XPS) are necessary for the elucidation of the enhancement mechanism of the ORR by caffeine on single crystal electrodes of Pt."

(Comment 6)

The authors described "The surface selection rule of IRAS indicated that caffeine is adsorbed on Pt(111) and Pt(110) with its molecular plane perpendicular to the surface above 0.1 V (vs. RHE), whereas it is adsorbed on Pt(100) with its molecular plane tilted relative to the surface above 0.5 V (vs. RHE)." To support this point, it is necessary to provide more visual and obvious evidence.

(Reply)

In-situ STM or FM-AFM is necessary for more visual and obvious evidences. It will take several years to obtain these evidences. Determination of surface morphology of caffeine modified Pt single crystal electrodes is our next target of our study.

Reviewers' comments:

Reviewer #1 (Remarks to the Author):

I do not think the authors carefully addressed my comments on the manuscript, neither for the comments from the other reviewers.
For example, Comment 1: The authors should read the reference RSC Adv., 2014,4, 52640-52646 carefully. It did reported the caffeine treated Pt/Au catalysts for ORR. There are also many other reports talking about the caffeine doped carbons for ORR. All these relevant work should be discussed in the introduction section.
Comment 2: The added sentences described the modification of caffeine on the electrolyte, not on Pt crystals.
Comment 3: I'm asking for the SEM images showing the morphology of the Pt electrodes, not the atomic and molecular images of Pt electrodes
Comment 4: The authors made the statement of "“No effects of caffeine on the ORR activity on Pt(100) also supports that the ORR activity of caffeine itself is negligible.” without any experiment results demonstration. There do have Some reports mentioned that caffeine itself also shows some ORR activity.
Comment 6: The authors claimed that The ORR activity of caffeine modified Pt(111) was still 6.2–fold as high as that without caffeine after 5000 cycles. However, no related experimental results are provided.

As a result, I do not think the manuscript is acceptable unless the authors carefully address all of my, and the other reviewers' comments by providing some additional experimental results or meaningful explanations, rather than simple statements without any support data.

Reviewer #2 (Remarks to the Author):

the authors have replied to most of my comments so only a couple of minor things:

1. I still think that Fig 1a red line has a (small) shift in the OHads butterfly peak of ca. 10-15 mV. Not only that but also the HUPD region is shifted in all three surfaces (for example in Pt(111), larger “double layer” for caffeine covered than for clean surfaces is found). I understand that the decrease in charge could be due to partially blocked surface with the caffeine, but why do the authors think that the potential regions shift in the presence of caffeine? Could it be due to changes in the interfacial pH? Maybe local surface charge? What do you think?
2. Page 5, new paragraph in yellow: typo in caffeine. It is written as “caffein” in the text.
3. Page 5, new paragraph: “Although Pt¬–OH is present in “the adsorbed hydrogen region”20, the adsorbed caffeine will block one electron transfer of the Pt–OH formation (Pt + H2O → Pt–OH + H+ + e–) as it prevents the one electron transfer due to the adsorption and desorption of Pt–H. ” That paper is about OHads detection in the HUPD in stepped surfaces, (see also Nature Energy 5 (11), 891-899 and Angewandte Chemie International Edition 56 (47), 15025-15029) not for Pt(111) where only Hads takes place below 0.3 V.

Reviewer #3 (Remarks to the Author):

The authors have revised the manuscript accordingly. It can be considered as a publication

Reply to the Reviewers' Comments for 2nd Version

Reviewer #1:

Thank you for your comments to improve our manuscript. Reply to the comments have been listed below. Revised parts have been highlighted in yellow in the manuscript.

(Comment 1)

The authors should read the reference RSC Adv., 2014,4, 52640-52646 carefully. It did reported the caffeine treated Pt/Au catalysts for ORR. There are also many other reports talking about the caffeine doped carbons for ORR. All these relevant work should be discussed in the introduction section.

(Reply)

Following sentences have been inserted in the last paragraph of page 3. References 19 and 20 have been added in the reference list.

"Caffeine is used as a capping agent and a structure directing agent for the preparation of well dispersed Pd–Au nanochain networks with unique structures, and enhances the ORR activity[20]. The ORR activity of caffeine derived graphene-wrapped Fe_3C nanoparticles is higher than that of Pt/C in alkaline solution[21]. However, the effects of caffeine itself on the ORR have not been examined on single crystal electrodes thus far."

20. Zhang, Q.-L., Feng, J.-X., Wang, A.-J., Weia, J. & Feng, J.-J. Simple synthesis of bimetallic alloyed Pd–Au nanochain networks supported on reduced graphene oxide for enhanced oxygen reduction reaction, *RSC Adv*. 4, 52640-52646 (2014).

21. Chen, M.-T., Huang, Z.-X., Ye X., Zhang, L., Feng, J.-J. & Wang, A.-J. Caffeine derived graphene-wrapped Fe3C nanoparticles entrapped in hierarchically porous Fe-N-C nanosheets for boosting oxygen reduction reaction, *J. Colloid Interface Sci*. 637, 216–224 (2023).

(Comment 2)

The added sentences described the modification of caffeine on the electrolyte, not on Pt crystals.

(Reply)

We did electrochemical and IRAS measurements in 0.1 M $HClO_4$ containing caffeine. Caffeine was modified on a single crystal electrode of Pt as soon as the electrode is dipped in the caffeine containing solution. Following sentence has been inserted in the second

paragraph of page 17:
"Caffeine is adsorbed on a Pt single crystal electrode as soon as the electrode surface is dipped in 0.1 M $HClO_4$ containing caffeine."

(Comment 3)
I'm asking for the SEM images showing the morphology of the Pt electrodes, not the atomic and molecular images of Pt electrodes.
(Reply)
We did all the experiments using single crystal electrodes of Pt with more than 3 mm in diameter. We did not use single crystals of nanoparticles.
Previous STM measurements verified the morphology of a single crystal surface of a noble metal does not change after the modification of organic molecule: the single crystal surface is kept atomically flat under the layer of the organic molecule (K. Itaya, *Prog. Surf. Sci.* 58, 121 (1998)). The surface structure of a Pt single crystal electrode affects the voltammogram sensitively in the adsorbed hydrogen region. Voltammograms in Fig. 3 without caffeine are identical with those reported previously; the single crystal electrodes of Pt examined are well-defined before and after the caffeine modification.
SEM measurement of a surface structure of a single crystal electrode of Pt is very difficult because the single crystal surface of Pt is roughened as soon as it is exposed air without potential control. After a Pt single crystal is annealed, it is cooled in Ar atmosphere down to room temperature. The surface of Pt single crystal electrode must be protected with ultrapure water during the transfer to an electrochemical cell. If we measure SEM of a single crystal electrode of Pt, we need an attachment box filled with Ar and transfer arm to the SEM apparatus. Furthermore SEM does not have a resolution to measure the adsorption geometry of caffeine. If the viewer care about the adsorption geometry of caffeine, in-situ STM or FM-AFM measurements are necessary. This is the next target of our study.

(Comment 4)
The authors made the statement of "No effects of caffeine on the ORR activity on Pt(100) also supports that the ORR activity of caffeine itself is negligible." without any experiment results demonstration. There do have Some reports mentioned that caffeine itself also shows some ORR activity.
(Reply)
We do not deny the ORR activity of caffeine itself in some conditions. However, no effect of caffein on Pt(100) (Fig. 4) shows "adsorbed caffeine itself on Pt electrodes" does

not affect the ORR of Pt electrodes. Following sentence has been added to the first paragraph of page 8.

“Caffeine itself might have the ORR activity. However, no effects of caffeine on the ORR on Pt(100) (Fig. 4) supports that the ORR activity of adsorbed caffeine itself is negligible on Pt electrodes.”

(Comment 6)

The authors claimed that The ORR activity of caffeine modified Pt(111) was still 6.2–fold as high as that without caffeine after 5000 cycles. However, no related experimental results are provided.

(Reply)

The durability of caffeine modified Pt single crystal electrodes is out of the scope of this paper, and will be reported in our forthcoming paper.

[Redacted to protect unpublished data]

Reviewer #2

Thank you for your comments to improve our manuscript. Reply to the comments have been listed below. Revised parts have been highlighted in yellow in the manuscript.

(Comment 1)

I still think that Fig 1a red line has a (small) shift in the OH_{ads} butterfly peak of ca. 10-15 mV. Not only that but also the H_{UPD} region is shifted in all three surfaces (for example in Pt(111), larger "double layer" for caffeine covered than for clean surfaces is found). I understand that the decrease in charge could be due to partially blocked surface with the caffeine, but why do the authors think that the potential regions shift in the presence of caffeine? Could it be due to changes in the interfacial pH? Maybe local surface charge? What do you think?

(Reply)

Nitrogen containing caffeine may change electronic state of the single crystal electrodes examined, giving the potential region shifts mentioned above. Following sentence has been inserted in the last paragraph of page 15.

"The change of the electric state due to the caffeine adsorption may also cause the potential shifts of the peaks of voltammograms in caffeine containing solution (Fig. 3)."

(Comment 2)

Page 5, new paragraph in yellow: typo in caffeine. It is written as "caffein" in the text.

(Reply)

We have corrected the typo. Thank you for the suggestion.

(Comment 3)

Page 5, new paragraph: ″Although PtOH is present in ″the adsorbed hydrogen region″ 20, the adsorbed caffeine will block one electron transfer of the PtOH formation ($Pt + H_2O \rightarrow PtOH + H^+ + e^-$) as it prevents the one electron transfer due to the adsorption and desorption of Pt–H. "

That paper is about OH_{ads} detection in the H_{UPD} in stepped surfaces, (see also Nature Energy 5 (11), 891-899 and Angewandte Chemie International Edition 56 (47), 15025-15029) not for Pt(111) where only H_{ads} takes place below 0.3 V.

(Reply)

We have revised the sentence in the last paragraph of page 5 as follows, and have added

the references the reviewer suggested.
"Although Pt–OH is present in "the adsorbed hydrogen region" of Pt(100), Pt(110) and stepped surfaces of Pt according to previous reports[22-26], the adsorbed caffeine will block one electron transfer of the Pt–OH formation ($Pt + H_2O \rightarrow Pt–OH + H^+ + e^-$) as it prevents the one electron transfer due to the adsorption and desorption of Pt–H. "

REVIEWERS' COMMENTS:

Reviewer #1 (Remarks to the Author):

The authors have addressed all of my comments and I would recommend the acceptance of the revised manuscript.
